# Enterocervical Fistula Six Weeks After Laparoscopic Mesh Sacral Colposuspension: A Case Report

**DOI:** 10.7759/cureus.80306

**Published:** 2025-03-09

**Authors:** Elyssa Marmolejo, Jason C Massengill

**Affiliations:** 1 Obstetrics and Gynecology, Medical College of Wisconsin, Milwaukee, USA; 2 Obstetrics and Gynecology, Wright State University Boonshoft School of Medicine, Dayton, USA; 3 Obstetrics and Gynecology, Wright Patterson Air Force Base, Dayton, USA

**Keywords:** cervix, colposuspension, enterocervical, fistula, mesh

## Abstract

Laparoscopic colposuspension with mesh is a common procedure performed to treat apical pelvic organ prolapse refractory to non-surgical interventions. Mesh complications involving a fistula are an uncommon event that typically occurs months or years later. We report a case complicated by enterocervical fistula that occurred six weeks after mesh placement. This was the case of a 67-year-old female with no surgical history who underwent a laparoscopic supracervical hysterectomy, bilateral salpingo-oopherectomy, laparoscopic colposuspension with mesh, and midurethral sling for treatment of stage II anterior and apical pelvic organ prolapse and stress urinary incontinence. She presented to the clinic for her six-week postoperative appointment complaining of vaginal discharge. Physical examination findings were significant for abnormal tissue protruding from the cervix. Examination under anesthesia revealed necrotic tissue within the cervical os. Subsequent laparoscopy revealed an enterocervical fistula resulting from small bowel adhering to the colposuspension mesh. This required cervical debridement, lysis of adhesions, partial mesh excision, and small bowel resection with primary anastomosis. We concluded that although rare, small bowel adhering to mesh can fistulize rapidly, and in this case presented as necrotic cervical tissue. While re-peritonealization has shown no significant difference in small bowel complication rates, the formation of a subacute fistula, in this case, highlights a rare complication. Treatment in the case involved an individualized approach weighing the risk of infection and the need for subsequent operation in leaving the remaining mesh versus the risk of bladder injury, hematoma, and vaginotomy in removing the entirety of the mesh.

## Introduction

Laparoscopic colposuspension with mesh is a common procedure to manage apical and anterior pelvic organ prolapse refractory to non-surgical interventions. Long-term success rates of mesh colposuspension are reported as 68.9-88.0% and it is generally considered the gold-standard method to treat advanced apical prolapse [1]. According to a meta-analysis, complications of prolapse repair can include mesh erosion occurring in 10.3% of cases, wound granulation occurring in 7.8%, and dyspareunia occurring in 9.1% [2]. Fistula formation following pelvic surgery, specifically hysterectomy, is uncommon with a reported rate of 0.1-4% where most of these involved the urinary tract [3]. Fistulas involving the bowel following pelvic surgery are exceedingly rare with only a few cases reported and most were associated with vaginotomy or bowel adhesiolysis/injury during the index surgery [4,5]. Here, we report a rare complication of enterocervical fistula occurring six weeks after the patient’s initial operation and presenting as asymptomatic vaginal discharge.

## Case presentation

A 67-year-old female with a history of stage II anterior and apical pelvic organ prolapse, stress urinary incontinence, and no previous abdominal surgeries underwent a laparoscopic supracervical hysterectomy, bilateral salpingo-oopherectomy, laparoscopic colposuspension with mesh, midurethral sling, and cystoscopy following one year of pessary therapy. Surgery was performed by a board-certified Urogynecology and Reconstructive Pelvic Surgeon. His standard practice involved utilizing a low-weight polypropylene mesh with six interrupted suture attachments on both the anterior and posterior walls utilizing a non-absorbable, braided polyester suture. His practice did not involve re-peritonealization of the mesh following placement. There were no pertinent intraabdominal findings, and the surgery involved no manipulation of the bowel. The immediate postoperative course was complicated by a brief episode of unresponsiveness 8 hours following surgery, which was determined to be a rare reaction to promethazine. She had no residual effects 12 hours following this episode.

On postoperative day 10, she complained of a new-onset bulge after feeling a “pop” when standing up from the couch. She had no complaints of pain, bleeding, or urinary or bowel changes. On exam, she was observed to have a new stage II distal posterior wall prolapse. The apex and anterior wall appeared well supported, and there were no other concerning findings on the exam.

**Figure 1:**
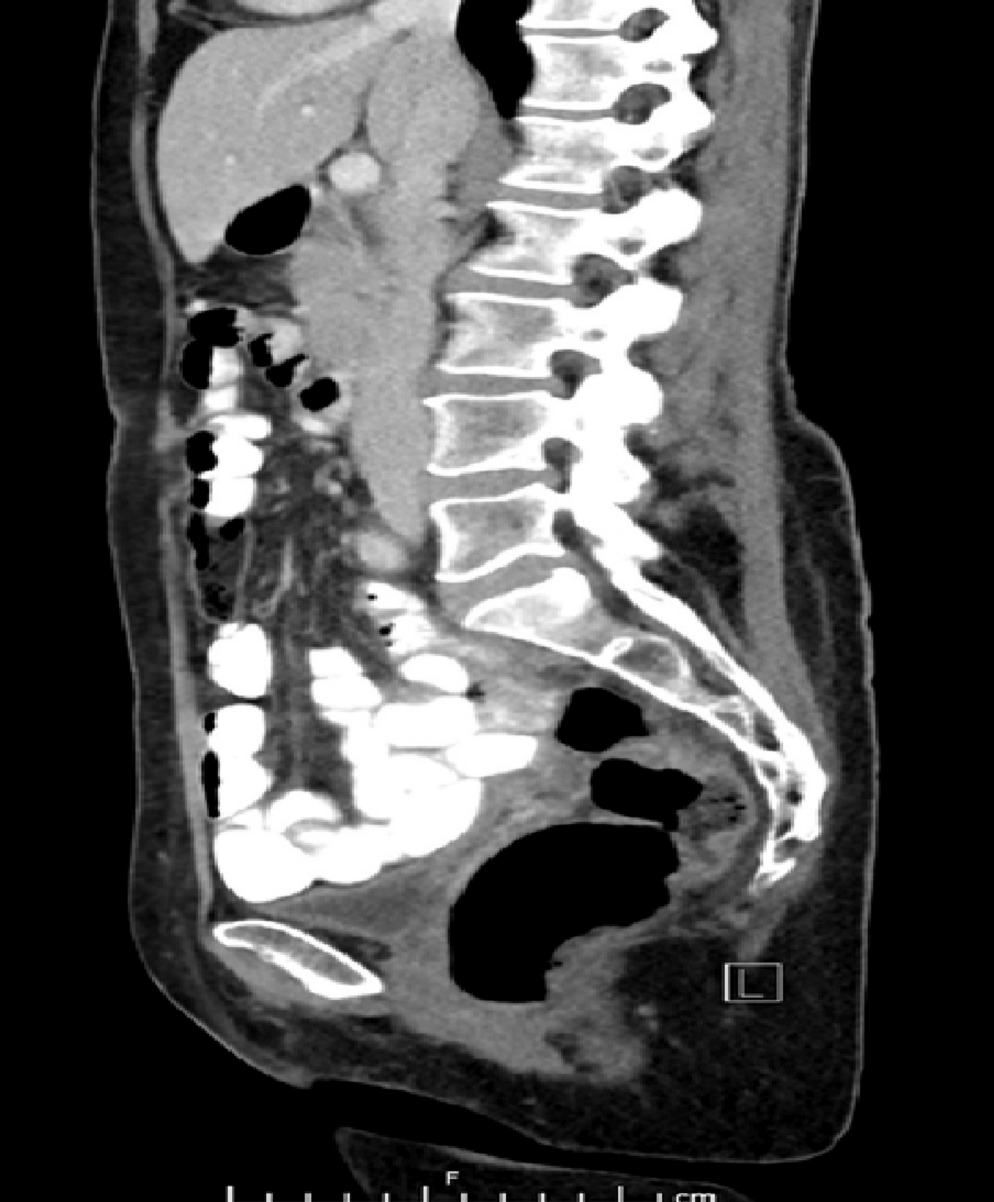
Midline sagittal cut of CT abdomen/pelvis

**Figure 2:**
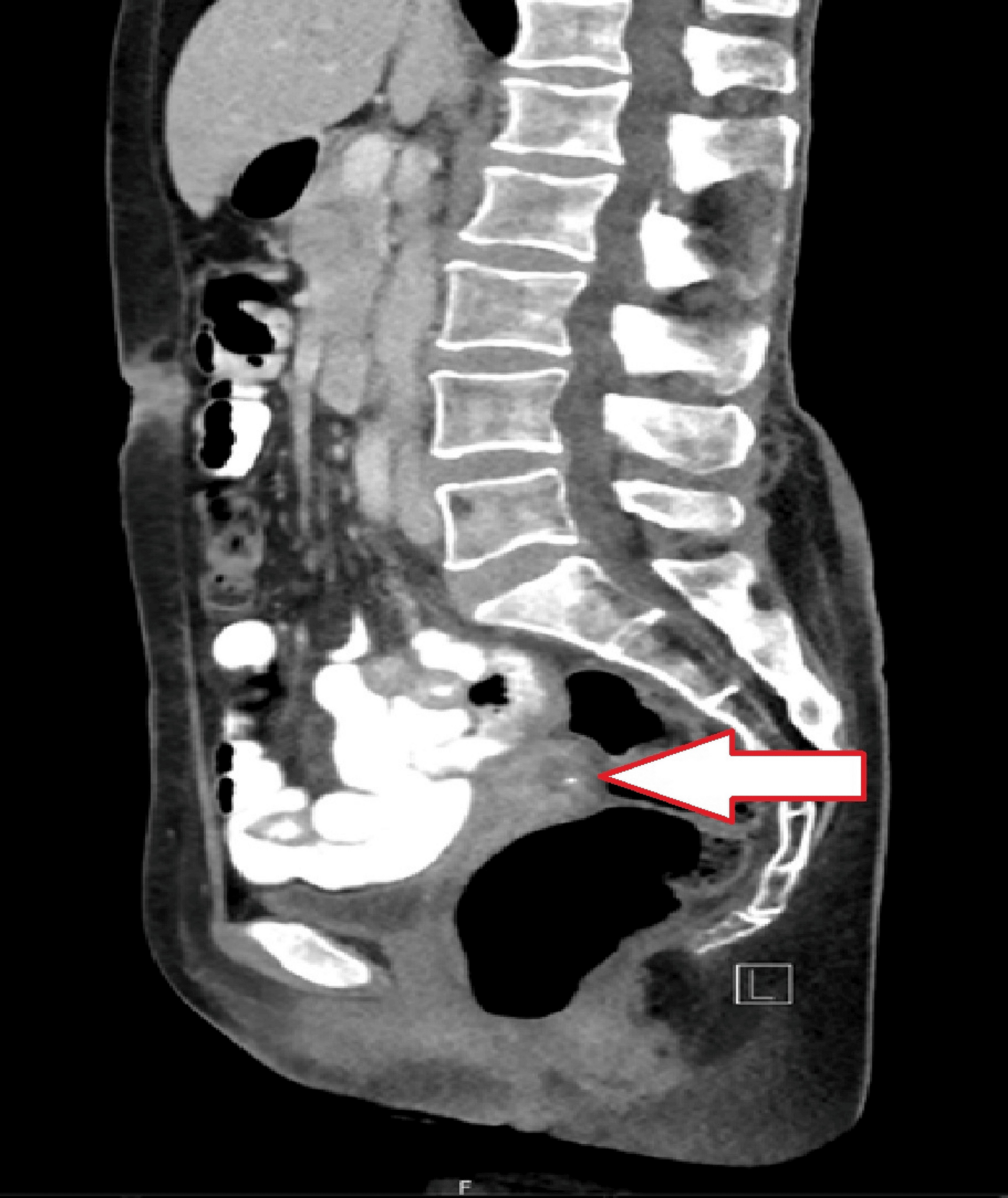
Left of the midline, sagittal cut of CT abdomen/pelvis showing a fluid collection

**Figure 3:**
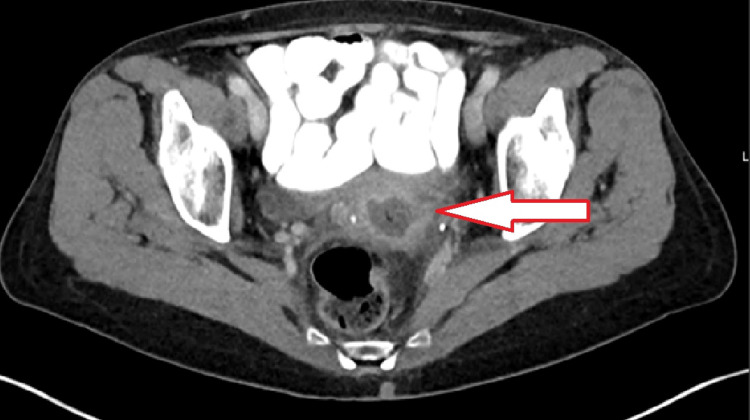
Axial cut of CT pelvis showing a fluid collection in cervix stroma

At her scheduled postoperative visit six weeks after surgery, the patient complained of abnormal brown vaginal discharge without odor. She denied fevers, chills, or pain. Her vital signs were normal. Physical examination revealed a prolapsing white mass with a bowel-like loop appearance coming from the internal os of the cervix. The patient was hemodynamically stable and the laboratory workup did not show an elevated white blood cell count. Computed tomography of the abdomen and pelvis with contrast were obtained (Figures 1-3) and revealed no obvious bowel communication to the vagina but did show a 2 x 2.7 x 2.7 cm fluid collection with air above and to the left of the cervix.

Given the unclear diagnosis and etiology, the patient was counseled for a return trip to the operating room for an exam under anesthesia and treatment. In the operating room, vaginoscopy and cystoscopy revealed a necrosing cervix without involvement of the bladder. No mesh was seen or palpable. The decision was then made to perform a repeat laparoscopy to investigate for etiology. Upon entering the abdomen, small and large bowel were adhered to the laparoscopic mesh well above the cervical attachment. Lysis of adhesions was performed. Once the small bowel was removed from the mesh, a small communicating fistula was found between the small bowel and the mesh. Bowel contents appeared to be communicating to the cervix via the mesh without evidence of infection present. General surgery was consulted for a small bowel resection with primary anastomosis which was performed through a 6 cm open suprapubic transverse laparotomy incision. During the case, the anterior and posterior laparoscopic mesh directly attached to the cervix that communicated with the bowel was excised. This involved the removal of most of the mesh bridge to the sacrum, although the retroperitoneal space was not entered to remove the mesh entirely. The mesh attached to the distal anterior and most of the posterior apical walls of the vagina were left in place. Cervical debridement was performed abdominally and vaginally leaving a thin circumferential rim of cervical tissue intact. 

Surgical pathology of the bowel reviewed no new findings explaining the fistula, specifically there was no evidence of Crohn’s disease or ulcerative colitis. Postoperatively, the patient had no further complications. One-and-a-half years after the fistula surgery, she continued to complain of mild bulge symptoms from her rectocele but declined further treatment. Physical examination revealed good support of the anterior and apical vaginal compartments without recurrence of apical prolapse or fistula.

## Discussion

Gynecologic fistula formation is rare. Risk factors for gynecologic fistula include a history of cesarean section, gynecologic carcinoma and/or radiation, uterine diseases such as endometriosis, pelvic inflammatory disease, infection, trauma, and other history of pelvic surgery [6]. The majority of genitourinary fistulas occur secondary to benign gynecologic procedures. This is likely related to the proximity of structures and organs in this region that can form an abnormal connection. Proposed mechanisms include direct tissue injury that is apparent or missed at the time of the index surgery, suboptimal use of surgical instruments, thermal injury, and suture placement that connects two organs [6]. Our case had no known intraoperative complications that would have raised our concern for a postoperative fistula and thus we do not truly know its origin. 

This case is unique in its clinical presentation and the decision-making required throughout management. She presented just six weeks following her operation with a sole complaint of non-malodorous, brown vaginal discharge. She had no evidence of infection and the tissue present at the cervix did not initially appear necrotic. Diagnosis of the enterocervical fistula through the mesh ultimately was found after a thorough laparoscopic investigation. Before colposuspension, this patient had no history of prior surgeries or any of the aforementioned risk factors that may have increased her risk for fistula formation. 

Surgical re-peritonealization at the time of this procedure was once standard practice; however, surgical re-peritonealization is likely not as beneficial as once hypothesized. One animal study showed the perineum heals quickly and physiologic re-peritonealization likely occurs within 24 hours without appreciable differences in adhesion patterns one month later [7]. Recent cohort studies comparing the complication rate in sacral colpopexy performed with and without re-peritonealization found that there was no significant difference in bowel complications or readmission rates among groups. Furthermore, re-peritonealization adds additional time to the procedure [8,9]. 

Following these reports, the common practice began to shift away from this approach, recognizing that colposuspension procedures could be performed in less time while also avoiding major structures, like the ureters, that could be injured in the re-peritonealization process. In this patient, the lack of re-peritonealization may have allowed enteric structures to adhere to the mesh and form a fistula in a relatively short time period. There are two case reports of fistula following similar procedures [4,5]. One of them reported an enterovaginal fistula following abdominal sacral colpopexy with minimal re-peritonealization during the index surgery. This complication occurred approximately 4-6 months after the index surgery [5]. This case report was written prior to the recent studies concluding an insignificant difference in complication rate with re-peritonealization.

Finally, an important decision point in this case was whether to take a mesh-preserving approach in the setting of a known bowel fistula. Leaving the mesh attached to the cervix and vagina posed the risk of infection and the need for a future corrective procedure, but removing it posed the risk of bladder injury, vaginotomy, recurrent prolapse, and recurrent fistula formation. Ultimately, the decision was made to leave the remaining mesh in place because the patient was hemodynamically stable, non-toxic appearing, and relatively asymptomatic despite complaining of abnormal discharge. Because of these factors, the infection risk appeared lower than the risks associated with removing the mesh. Following the operation, she was treated with broad-spectrum antibiotics for several days and had an uncomplicated postoperative course.

## Conclusions

This challenging case highlights an early enterocervical fistula from the bowel through laparoscopic colpopexy mesh applied to the cervix and anterior/posterior vaginal walls in a patient with no prior history of surgeries or other risk factors besides her initial operation. It also demonstrates an early fistula with a relatively non-concerning clinical presentation. Further analysis of the case posed the question of whether re-peritonealization may have prevented this particular complication, despite recent studies concluding that there was no difference in complication rate with or without this intervention. The intraoperative decision to take a mesh-preserving approach was individualized with a good outcome for the patient.

## References

[1] Hussain U, Kearney R (2013). Surgical management of stress urinary incontinence. Obstet Gynaecol Reprod Med.

[2] Abed H, Rahn DD, Lowenstein L, Balk EM, Clemons JL, Rogers RG (2011). Incidence and management of graft erosion, wound granulation, and dyspareunia following vaginal prolapse repair with graft materials: A systematic review. Int Urogynecol J.

[3] Forsgren C, Altman D (2010). Risk of pelvic organ fistula in patients undergoing hysterectomy. Curr Opin Obstet Gynecol.

[4] Mickelson L, Miklos JR, Moore RD (2016). Laparoscopic repair of enterocervical fistula after mesh erosion into the sigmoid colon and cervix after robotic supracervical hysterectomy and sacrocervicopexy. Female Pelvic Med Reconstr Surg.

[5] Hopkins MP, Rooney C (2004). Entero mesh vaginal fistula secondary to abdominal sacral colpopexy. Obstet Gynecol.

[6] Abrams M, Pope R (2021). Obstetric and gynecologic genitourinary fistulas. Clin Obstet Gynecol.

[7] Nazik H, Narin MA, Narin R, Dağlıoğlu K, Ünal Ï, Tap O, Aytan H (2014). Investigating the clinical significance of mesh peritonization in abdominal vault suspension surgery using a comparative rabbit model. Eur J Obstet Gynecol Reprod Biol.

[8] Glass Clark SM, Shannon MB, Gill E, Clark MD, Lamb E, Carroll A (2020). Complications after reperitonealization of mesh at time of sacrocolpopexy: A retrospective cohort study. Female Pelvic Med Reconstr Surg.

[9] Kulhan M, Kulhan NG, Ata N, Nayki UA, Nayki C, Ulug P, Yilmaz N (2018). Should the visceral peritoneum be closed over mesh in abdominal sacrocolpopexy?. Eur J Obstet Gynecol Reprod Biol.

